# Physiology versus evidence-based guidance for critical care practice

**DOI:** 10.1186/cc14725

**Published:** 2015-12-18

**Authors:** Luciano Gattinoni, Eleonora Carlesso, Alessandro Santini

**Affiliations:** 1 Dipartimento di Fisiopatologica Medico-Chirurgica e dei Trapianti, Università degli Studi di Milano, via Francesco Sforza 35, 20122 Milan, Italy; 2 Dipartimento di Anestesia, Rianimazione ed Emergenza Urgenza, Fondazione IRCCS Ca' Granda - Ospedale Maggiore Policlinico di Milano, via Francesco Sforza 35, 20122 Milan, Italy

## Abstract

Evidence based medicine is an attempt to optimize the medical decision process through methods primarily based on evidence coming from meta-analyses, systematic reviews, and randomized controlled trials ("evidence-based medicine"), rather than on "clinical judgment" alone. The randomized trials are the cornerstones of this process. However, the randomized trials are just a method to prove or disprove a given hypothesis, which, in turn, derives from a general observation of the reality (premises or theories). In this paper we will examine some of the most recent randomized trials performed in Intensive Care, analyzing their premises, hypothesis and outcome. It is quite evident that when the premises are wrong or too vague the unavoidable consequences will be a negative outcome. We should pay when designing the trial an equal attention in defining premises and hypothesis that we pay for the trial conduction.

## Introduction

It is not easy to state when and where intensive care medicine was born. Although continuous assistance, as provided during the Crimean War by Florence Nightingale, included the basic principles of intensive care, most people agree that the Copenhagen polio epidemic represents the starting point of this discipline. Establishing respiratory homeostasis, buying time for recovery through artificial ventilation, still represents one of the basic principles on which intensive care is founded.

The substantial difference between intensive care and other medical disciplines is that the latter aim at correcting and treating specific diseases, while intensive care primarily treats the homeostasis of the whole person. In general, while most diseases originate in the domain of molecular biology, the homeostasis is maintained by bulk flows of gas and blood at appropriate pressures, which are in the domain of physiology. Therefore it has been natural that intensive care and physiology since the very beginning were strictly associated.

In the mid-1980s Alvin Feinstein [1] and David Sackett et al. [2] translated epidemiological methods into physician decision-making, in an attempt to optimize the medical decision, based primarily on evidence coming from meta-analyses, systematic reviews, and randomized controlled trials ("evidence-based medicine"), rather than on "clinical judgment" alone. Although Sackett et al. explicitly stated that this approach necessitates "integrating individual clinical expertise with the best available external clinical evidence from systematic research" [3], in the following years the systematic research, primarily from randomized trials, became the only accepted way to assess the value of a given intervention. This approach led to the flowering of new professional figures, as "meta-analysts" and "trialists", who had the final word on judging the internal validity (how it is performed) and the external validity (how it can be generalized) of a given trial. The results of "good trials", according to the adherents to the new "religion" of evidence-based medicine, were incorporated in guidelines and recommendations. This process appears unquestioned and unstoppable. To rephrase a famous Bob Dylan line, there are no truths outside the gates of evidence-based medicine.

## The scientific method

**Figure 1:**
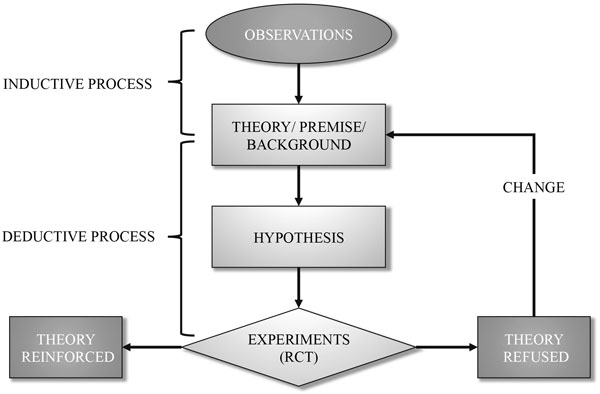
**Steps in the process of scientific knowledge accumulation**. *RCT *randomized controlled trial.

Epistemology is a word indicating the philosophy of science. Over the centuries, scientific reasoning has been developed according to quite definite pathways. Accordingly we may consider the place of randomized trials, currently the primary sources of evidence, in the mainstream of processes that allow the acquiring of knowledge [4]. Briefly, the process in medicine starts with empiric observations from which a theory or premise is developed. As an example, observing that an imbalance between oxygen supply and oxygen demand may lead to organ dysfunction, which in turn may be associated with increased mortality, led to a general theory that tissue hypoxia is harmful. Physiological reasoning showed that central venous oxygenation (SvO_2_) is an indicator of the balance between oxygen supply and demand. In intensive care, therefore, an accepted theory/premise is that oxygen imbalance is harmful and that SvO_2 _may measure it. The theories therefore follow an inductive process: from particular observations it is possible to derive a general statement. Until the 17th century the validity of the theories had to be found inside the mind, as for Aristotle or Descartes, or founded on God, as in Saint Thomas in the Middle Age. From the general law, through a logic process, a deduction was made. This way of reasoning is the structure of the syllogism: "All men are mortals. Socrates is a man. Therefore, Socrates is mortal". What dramatically changed in the 17th century was the approach to validate the theories from which hypotheses were generated. The theory was valid only if verified through an experiment and described in mathematical language, as for Galileo and Newton. In the 20th century Popper reasoned that it is impossible to prove that a given theory is valid; it is only possible to prove that it is incorrect. This is the basis of the trial and error approach (disprove a theory and generate a new one). Note, however, that the deductive process, through which from a general law a particular hypothesis is derived, has been unquestioned throughout the centuries. A summary of the steps required to improve our scientific knowledge is presented in Figure 1.

## Premises disproved by trials: the supranormal hemodynamics example

Randomized trials are one of the possible experiments which can be used to prove or disprove a given hypothesis that, in turn, originates from a given theory. If tissue hypoxia is harmful and SvO_2 _is one of the forms of its measurement (inductive reasoning), a possible hypothesis is that its correction while monitoring/targeting SvO_2 _may decrease the organ dysfunction and improve survival (deductive reasoning). It is worth noting that "the truth", which the trial aims to disclose, is implicit in the premises/theories and that a "negative" trial just indicates that the premises are incorrect or incomplete.

The tissue hypoxia theory generated a series of trials, starting with Shoemaker et al. [5] who proposed supranormal values of the cardiac index to improve survival. The implicit theory/premise was that critically ill patients have a supranormal need for oxygen, implying that a normal supply of oxygen would not be sufficient for their needs. Firm physiological support was lacking, however, as the Shoemaker theory derived simply from the observation that patients who survived after major surgery had higher cardiac indexes than the patients who died. This theory was then translated, without solid physiological plausibility, to the entire population of intensive care patients. Two randomized trials failed to show differences between the patients treated with supranormal values and normal values [6,7] and this was the end of targeting supranormal hemodynamic variables in general intensive care. These two studies showed that the premises were incomplete/wrong; that is, there is no necessity for a greater global oxygen supply in critically ill patients.

In one of these studies [7], however, another arm, apart from the supranormal arm, was added. In this group of patients the treatment was directed to reach/maintain SvO_2 _at values >70%. Results showed that this group was indistinguishable from a group in which normal values of the cardiac index (>2.5 l/minute/m^2^) were the aim of treatment, indicating that targeting the hemodynamic treatment to normal SvO_2 _or to normal cardiac index are equivalent strategies. Of note, however, the SvO_2 _values recorded in all patients entering into the study were close to the target of 70%, indicating that oxygen imbalance, on average, was absent in the general ICU population studied.

## Wrong premises versus wrong conclusions: the early goal-directed therapy trials example

Ten years later, in a subgroup of critically ill patients with severe sepsis and septic shock, Rivers et al. [8] presented a study of early goal-directed therapy (EGDT). The premises/theories behind the study were that: oxygen imbalance is common in patients with severe sepsis and septic shock; hypoxemia leads to multiorgan dysfunction and mortality; and SvO_2 _is the indicator of the oxygen supply/demand balance. The hypothesis deduced from these premises and tested by Rivers et al. was that an early intervention, aimed at correcting the oxygen supply/demand imbalance, could improve survival by limiting organ dysfunction. Actually, the Rivers et al. study showed an impressive improvement in survival in the EGDT group. Note that a baseline SvO_2 _of around 50% in the patients enrolled clearly indicated the presence of an oxygen imbalance.

To improve the evidence and to dissipate some concerns about the Rivers et al. study, three other large trials were organized in the following years: the Protocolised Management in Sepsis [ProMISe] trial [9], the Australasian Resuscitation in Sepsis Evaluation [ARISE] trial [10] and the Protocolized Care for Early Septic Shock [ProCESS] trial [11]. Despite some differences in trial design, the core of these experiments was the same: to test whether EGDT, as designed by Rivers et al., is of real benefit in patients with severe sepsis or septic shock. All trials tested the same premises/theories that Rivers et al. tested but, in contrast to their results, no outcome benefits were found in the treated groups. The conclusions were that EGDT in sepsis and septic shock does not work. What these trials actually proved was simply that one or more of the theories/premises on which the trials were based was false. In observing the population enrolled in these trials, it is quite obvious that the first premise ("oxygen imbalance is common in patients with severe sepsis and septic shock") was false. Actually the average SvO_2 _at baseline was ≥70% in all the three trials, compared with 50% in the Rivers et al. study. Therefore, in our opinion, these trials simply showed that the oxygen imbalance may not be common in the sepsis/septic shock population as currently defined. Not surprisingly, any intervention to correct a nonexistent "oxygen imbalance" is futile. Consequently, to say that SvO_2 _measurement is useless, as possibly suggested by these trials' results, is nonsense. Physiology continues to tell us that SvO_2 _is an indicator of the oxygen supply/demand balance and that, when tissue hypoxia is really present - as in the Rivers et al. study [8] and not in the aforementioned recent trials [9-11] - the correcting intervention makes sense.

This is one example of how randomized trials may be misleading. There are many published trials in which the premises are so vague or biologically implausible that the so-called negative results are an unavoidable consequence.

## Strong premises and integration of physiology and evidence: the low tidal volume in acute respiratory distress syndrome example

Let us now examine what is probably the most important randomized clinical trial in intensive care: the lower versus higher tidal volume treatment in acute respiratory distress syndrome (ARDS) [12]. The premises of the trial were that nonphysiological stresses and strains applied by mechanical ventilation may produce further injury to the lung, which leads to an increase in mortality. Following experts in the field, lower tidal volume was set at 6 ml/kg ideal body weight (IBW) and higher tidal volume at 12 ml/kg IBW. Note that the IBW was kept as a surrogate of the normal lung dimensions expected in each individual. The results were straightforward: 6 ml/kg IBW ventilation provided clear-cut outcome advantages. From a different point of view, 12 ml/kg IBW provided clear-cut disadvantages. It must be noted, however, that previous studies comparing different values of tidal volumes such as ≅7 ml/kg vs. ≅10 ml/kg [13-15] did not find any difference. In addition, in some patients in which 6 ml/kg tidal volume induces hypoventilation, there is high possibility of reabsorption atelectasis, further hypoxemia, and right ventricular dysfunction. Therefore while the "evidence" tells us that 12 ml/kg is more dangerous than 6 ml/kg, only the physiology may tell us whether 6 ml/kg is adequate or "too high" [16], in which case extracorporeal respiratory support is indicated, or "too low" [17], in which case higher tidal volume may be required. Only the physiology-oriented approach may tell us to monitor and treat a patient according to his/her actual needs. In a sense, this is what was suggested by Sackett et al.: integrating clinical judgment (physiological based) with evidence (higher strain is in generally worse than lower strain).

## Strong physiological rationale without evidence: the positive end-expiratory pressure in ARDS example

The best example of how physiology overcomes the "evidence" is the setting of positive end-expiratory pressure (PEEP). PEEP at an undefined pressure level being of benefit in ARDS derives from a series of experimental and clinical studies. In normal lungs, as sustained by Webb and Tierney [18] in their famous experiment, the use of PEEP may prevent or sharply decrease the lung edema induced by "lethal tidal volume". These observations were confirmed by a long series of experiments which showed the "protective effect" of PEEP, particularly (or exclusively) if associated with a coincident lowering of tidal volume [19,20]. The clinical use of PEEP dates back to 1938, when Barach et al. [21] published the effects of PEEP on normal man and in patients with pulmonary edema. After Gregory et al.'s [22] observation in infants and the description of ARDS by Ashbaugh et al. [23], the use of PEEP in adults became widespread. Since then, PEEP has always been used in ARDS, aiming to improve oxygenation in the early years and to reduce lung injury by increasing lung homogeneity and avoiding intratidal collapse in the last decades [24]. Although the use of PEEP is unquestioned, its correct setting lays still in the darkness. If PEEP worked in ARDS as in experimental animals, there is no doubt that clinical results would be excellent. Unfortunately, when randomized controlled trials were performed, no clear-cut difference between higher and lower PEEP was observed. In fact, three studies in which PEEP was selected according to different methods, but ended up with a higher PEEP around 13-15 cmH_2_O and a lower PEEP around 7-9 cmH_2_O, showed similar mortality in the two arms [25-27]. These studies enrolled ARDS patients whose severity spanned from mild to severe according to the Berlin classification [28].

Let us now examine the premises and theories for which higher or lower PEEP should lead to differences in mortality: in ARDS, mechanical ventilation may be injurious as potentially producing intratidal collapse and decollapse, which is harmful to the lung structure; intratidal collapse and decollapse is usual in most ARDS patients; lower PEEP, although providing viable oxygenation, does not prevent injurious intratidal collapse and decollapse; and higher PEEP, although overdistending some lung regions and interfering more with hemodynamics, prevents injurious intratidal collapse and decollapse.

The hypothesis generated from these premises, which derive from physiological observations, is that in most ARDS patients the potential damages of higher PEEP are lower than the potential damages of intratidal collapse and decollapse, leading to less ventilator-induced lung injury and better outcome.

All trials comparing higher versus lower PEEP were "negative", indicating that the premises were incomplete or wrong. Physiology and commonsense tell us that for PEEP to work some recruitable collapse must be present. It must be noted that mild ARDS and a consistent fraction of moderate ARDS do not present collapsed regions which are recruitable within the range of conventional tidal volumes [29]; that is, there are no pre-requisites for PEEP to work.. Physiology tells us also that the more dependent the collapsed region, the higher the pressure required to keep that region open. Knowing this, it becomes evident that what the aforementioned randomized trials told us was simply that the second premise ("intratidal collapse and decollapse is usual in most of ARDS patients") is not satisfied. Therefore, to test whether the benefits of higher PEEP overcome its possible harms, compared with lower PEEP, patients in the trial should satisfy the second premise; that is, they must present collapsed/recruitable lung units, which are more frequent in severe ARDS. Actually some meta-analyses already suggest that the more severe the patient, the better the potential for a beneficial effect from higher PEEP [30]. A recent prospective trial that targeted such patients corroborates this interpretation [31].

## Conclusions

As currently practiced, evidence-based medicine relies on randomized controlled trials, from which reviews and meta-analyses primarily derive. It is evident that if the premises of the trials are incomplete, wrong, or too vague, the results of the trial will be negative. It is impressive to see the disproportion between the attention paid to the design of a trial compared with that spent analyzing the validity of the premises and the derived hypotheses. Negative results are often attributed to the heterogeneity of the population. This just derives from the lack of a clear definition of the problem that the trial is designed to solve. Severe sepsis/septic shock is by definition heterogeneous, but if we concentrated on the oxygen balance for patient selection then the heterogeneity problem would be in part overcome. ARDS is by definition heterogeneous, but if we concentrate on the amount of recruitability to enroll the patients in a PEEP trial then the heterogeneity would in part disappear. In conclusion, physiology helps to design premises and theories; without strong premises and theories, whatever the trial will prove is, in the best case, useless or, if misinterpreted, dangerous.

## Abbreviations

ARDS, Acute respiratory distress syndrome; EGDT, Early goal-directed therapy; IBW, Ideal body weight; PEEP, Positive end-expiratory pressure; SvO_2_, Central venous oxygenation.

## Competing interests

The authors declare that they have no competing interests.

## Notes

This article has been published as part of *Critical Care *Volume 19 Supplement 3, 2015: Future of Critical Care Medicine (FCCM) 2014. The full contents of the supplement are available online at http://www.ccforum.com/supplements/19/S3. Publication of this supplement was supported by Fresenius Kabi.
